# Implantoplasty combined with soft tissue grafting for the management of complex cases: A microsurgical approach

**DOI:** 10.1002/cap.70037

**Published:** 2026-04-15

**Authors:** João Batista César Neto, Rafael Lazarin, Henrique Rinaldi Matheus, Emerson Santiago, Giuseppe A. Romito

**Affiliations:** ^1^ Division of Periodontology College of Dentistry University of São Paulo São Paulo Brazil

**Keywords:** autografts, case series, dental implants, microsurgery

## Abstract

**Background:**

The treatment of peri‐implantitis and/or peri‐implant soft tissue dehiscence defects (PSTD) remains challenging. The incorporation of the operating microscope (OM) into surgical procedures might have changed the treatment landscape for such conditions around implants. The aim of this case series is to present three clinical cases in which the combination of connective tissue grafting (CTG) and implantoplasty was performed under microscopy and served as the key tools for managing complex cases.

**Methods:**

In this article, the authors illustrate three distinct cases in which the combination of CTG and implantoplasty was used as a single or double‐staged approach for the treatment of PSTD, combined or not with peri‐implantitis. Importantly, the OM was used as a critical tool to increase precision and reduce trauma.

**Results:**

Overall, all procedures successfully preserved implants up to 3 years in highly compromised conditions. The approaches achieved a reduction in mucosal recession of up to 2 mm and successfully increased the thickness of the soft tissues. In addition to the maximum precision combined with the lesser trauma, the OM was critical for allowing delicate flap elevation of extremely thin tissues, achieving the smoothest implant surface with minimal wear, and permitting intimate adaptation of the CTG to the recipient bed.

**Conclusion:**

The OM combined with advanced skills for surgeons facilitated critical steps of the surgical procedures. Microsurgery combined with implantoplasty and connective tissue grafting offers an effective alternative for managing supraosseous or exposed implant threads caused by peri‐implant disease or poor implant positioning.

**Key points:**

- Limited evidence supports implantoplasty with connective tissue grafting for complex implant complications, especially in esthetic areas.
- This case series shows implantoplasty aids decontamination and also reduces buccal implant volume, while connective tissue grafting enhances peri‐implant soft tissue thickness.
- The microsurgical approach was fundamental to treatment success. It improved precision, minimized trauma, and promoted better healing and lower morbidity.

**Plain language summary:**

This group of cases shows that using a combination of implant surface polishing (implantoplasty) and soft tissue grafts, done with a precise microsurgical technique, can be an effective and less invasive way to treat complicated cases, without needing to remove the implant.

## INTRODUCTION

Implantoplasty was introduced as a technique to smooth implant threads and simultaneously detoxifying the implant surface for the treatment of peri‐implantitis.^1^, ^2^ A recent meta‐analysis identified implantoplasty as effective in reducing probing depth, bleeding on probing, and/or suppuration.^3^ Since its introduction, implant fracture after implantoplasty has been a concern for clinicians. However, the literature indicates that such occurrences are rare,^4^, ^5^ supporting implantoplasty as an adjunctive tool for managing other challenging conditions beyond peri‐implantitis. This is particularly relevant when using an optical microscope that significantly enhances procedural precision. Multiscale structural dimensions found in dental implants, such as pitch distance (0.5–1 mm), peaks and valleys of 0.2–0.5 mm depth, and average roughness of 1–2 µm^6^ may compromise visualization by naked eye, and magnification might overcome this limitation in implantoplasty.

In buccally malpositioned teeth, root‐coverage techniques are challenging, and complete root coverage is unpredictable.^7^ In such cases, odontoplasty is performed to minimize these discrepancies and to reduce the avascular surface of the recipient bed.^8^ Interestingly, implants positioned buccally have a threefold higher risk of peri‐implant soft tissue dehiscence/deficiency (PSTD),^9^ and their position is a critical consideration in selecting the therapeutic strategy.^10^ Given the similarities across fields, implantoplasty may be used for reshaping buccal discrepancies around implants rather than solely for decontamination.

Significant evidence supports the use of autologous soft tissue grafting for the correction of PSTD,^10^ especially for managing PSTD after crown delivery, when predictability is decreased and the risk of complications increases.^11^ Although the definitive benefit of soft tissue augmentation procedures compared to, or in addition to, hard tissue regeneration for improving the short‐ and long‐term outcomes of dental implant therapy remains uncertain, some evidence suggests that soft tissue procedures may potentially reduce or eliminate the need for hard tissue augmentation around dental implants.^12^ The group 2 from the last EAO Consensus Conference compiled evidence indicating that soft tissue augmentation procedures may improve clinical parameters (gingival index, mucosal recession) and plaque control.^13^ In addition to the benefits provided by connective tissue grafts (CTG), the use of microsurgical approaches and magnification has been suggested to enhance the outcomes and predictability of soft tissue procedures around teeth.^14^, ^15^, ^16^, ^17^ When compared to conventional macroscopic techniques, microsurgical approaches, combined with magnification ranging from 2.5× to 30×, have been associated with higher rates of root coverage,^18^, ^19^, ^20^, ^21^, ^22^, ^23^ complete root coverage,^18^, ^19^, ^20^, ^23^, ^24^ and improved patient‐reported outcomes.^20^, ^22^, ^23^, ^25^ Although the literature on implant dentistry is still limited,^26^ similar benefits may be expected.

Thus, the aim of this case series is to present three clinical scenarios in which the combination of CTG and implantoplasty served as the key tools for managing complex cases, with the assistance of an operating microscope (OM) being fundamental to the execution of the proposed approaches.

## MATERIALS AND METHODS

This retrospective case series comprises three non‐consecutive implant complication cases treated in a private practice and reported in accordance with the PROCESS guidelines.

### Preoperative patient and site management

All patients were non‐smokers and systemically healthy adults (ASA I), who presented with soft tissue deficiencies around implants in the anterior region. Two cases also exhibited peri‐implant pockets.

Before surgery, patients were informed about the procedures, materials, medications, expected outcomes, potential complications, side effects, and alternative treatment options. Each patient provided oral consent before undergoing surgery, which was performed by an experienced periodontist (JBCN) in a private practice in Sorocaba, Brazil, between May 2022 and August 2024.

Additionally, all patients received necessary periodontal treatment and oral hygiene instructions prior to surgery. Surgery was scheduled only when oral hygiene was deemed satisfactory, defined as an overall plaque index (PI) and bleeding on probing (BOP) below 20%, with a PI of zero at the surgical site. Baseline clinical parameters were recorded on the day of surgery. A UNC‐15 periodontal probe was used to measure PI, BOP, probing depth (PD) in millimeters (mm) in six sites per implant, peri‐implant mucosa recession (MR) in mm (considering implant shoulder as reference), and keratinized tissue width (KTW) in mm at the center of the buccal aspect. These parameters were measured at the baseline and at the most extended follow‐up after surgery.

All patients in this case series received a CTG harvested from the palate with parallel incisions,^27^ 2 mm apart from each other. The epithelial layer was extraorally removed, and the grafts were trimmed to a thickness of approximately 1.5 mm. The dimensions of the grafts followed the dimensions of each recipient bed and will be described individually in each case. The donor sites received a collagen sponge for hemostasis and protection, and were closed with “X” sutures anchored around the teeth close to the site. Single interrupted sutures were used according to the needs of each region. All cases were treated using an OM (Advanced Decius Microscope, DF Vasconcelos) with magnification between 5× and 13×.

### Case 1

**FIGURE 1:**
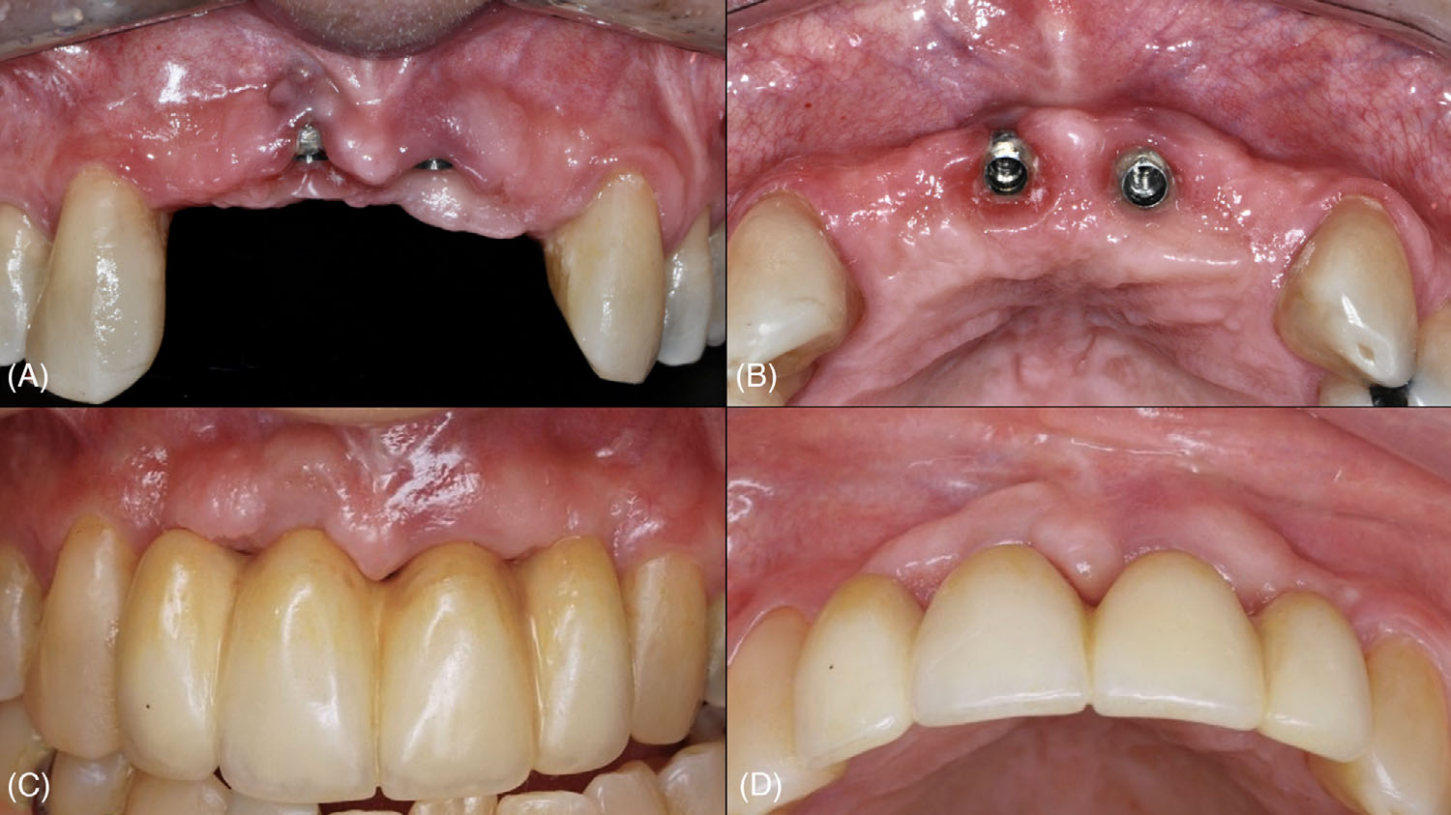
Case 1: Pre‐ and postoperative aspects of the surgical site. (A) Buccal view of the baseline defect. (B) Occlusal view of the baseline defect. (C) Buccal view at 3 years postoperatively. (D) Occlusal view at 3 years postoperatively.

**FIGURE 2:**
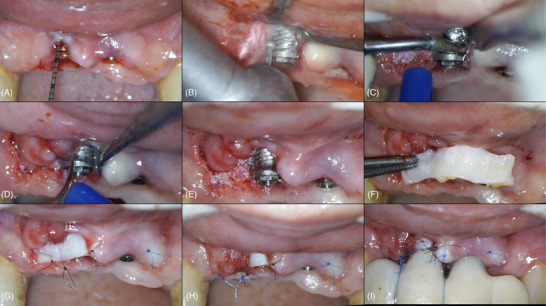
Case 1: Surgical procedure. (A) Probing. (B) Implantoplasty with spherical diamond bur. (C) Implantoplasty with an ultrasonic diamond tip of higher‐grain size. (D) Refinement of the implantoplasty with a pure metallic ultrasonic tip. (E) Final aspect of the implantoplasty. (F) Dimensions of the autologous CTG on the recipient bed. (G) Insertion and stabilization of the CTG inside the tunnel and stabilization of the CTG to the palatal bed. The graft was secured with a horizontal mattress 6.0 nylon suture that began on the palatal side, passed through the graft at two points, and returned to the palatal side. Single interrupted 7‐0 nylon sutures were then used to stabilize the graft to the palatal soft tissue at the edentulous ridge and close to the papilla between the implants. (H) Stabilization of the buccal flap. (I) Final aspect of the surgery, with the screwed provisional in position and suspensory sutures. These sutures originated on the buccal side, passed through the buccal flap and the graft, and over the contact point of the provisional restoration were placed at the mesial and distal aspects of the implant corresponding to tooth #8. Then, 7‐0 nylon sutures were used to secure the CTG to the buccal flap.

A 51‐year‐old female presented with esthetic concerns, impaired oral hygiene, and halitosis. Clinical examination revealed a soft tissue defect on the buccal aspect of the implant at tooth #8 and PD of 2 mm at the center of the buccal aspect, with exposed implant shoulder and threads, thin tissue, and dark mucosal discoloration. Tooth #9's implant shoulder was also shallowly positioned (Figure 1A,B). Treatment involved a microscope‐assisted soft tissue graft and implantoplasty (Figure 2A–I). A small vertical releasing incision was made near tooth #6, followed by a crestal incision to access tooth #8, and a tunnel preparation was extended to the buccal region of the implant at tooth #9. Following flap elevation, implantoplasty was performed to smooth the exposed implant threads, initially using a high‐speed spherical diamond bur (FG 1016, KG Sorensen), and subsequently employing a sequence of ultrasonic diamond and metallic tips (E7D Helse Ultrasonic, E6D Helse Ultrasonic, P10 Helse Ultrasonic) as suggested by the literature^28^ (Figure 2B–E). In order to minimize the retention of titanium particles in the surgical area, throughout the entire procedure, care was taken to ensure constant and adequate irrigation, as well as effective suction of the surgical site. Upon completion of the implantoplasty procedure, 24% ethylenediaminetetraacetic acid (EDTA) (Straumann PrefGel) was applied to the implant surface for 2 minutes to aid decontamination and remove residual debris, followed by thorough rinsing with saline solution.^29^ A CTG was harvested from the palate (Figure 2F) and stabilized with 6‐0 (Nylon Blue, Techsuture) and 7‐0 nylon (Nylon Black, Techsuture) sutures (Figure 2G,H). The graft was inserted through a tunnel, fixed with horizontal mattress and interrupted sutures, and additionally stabilized with buccal‐to‐palatal transfixation sutures. 7‐0 nylon (Nylon Black, Techsuture) sutures stabilized the CTG to the buccal flap, as recommended by the literature.^30^, ^31^ The provisional restoration was screwed in, and sutures secured the CTG to the buccal flap (Figure 2I). More surgical details are available in the online video (Video S1).

**FIGURE 3:**
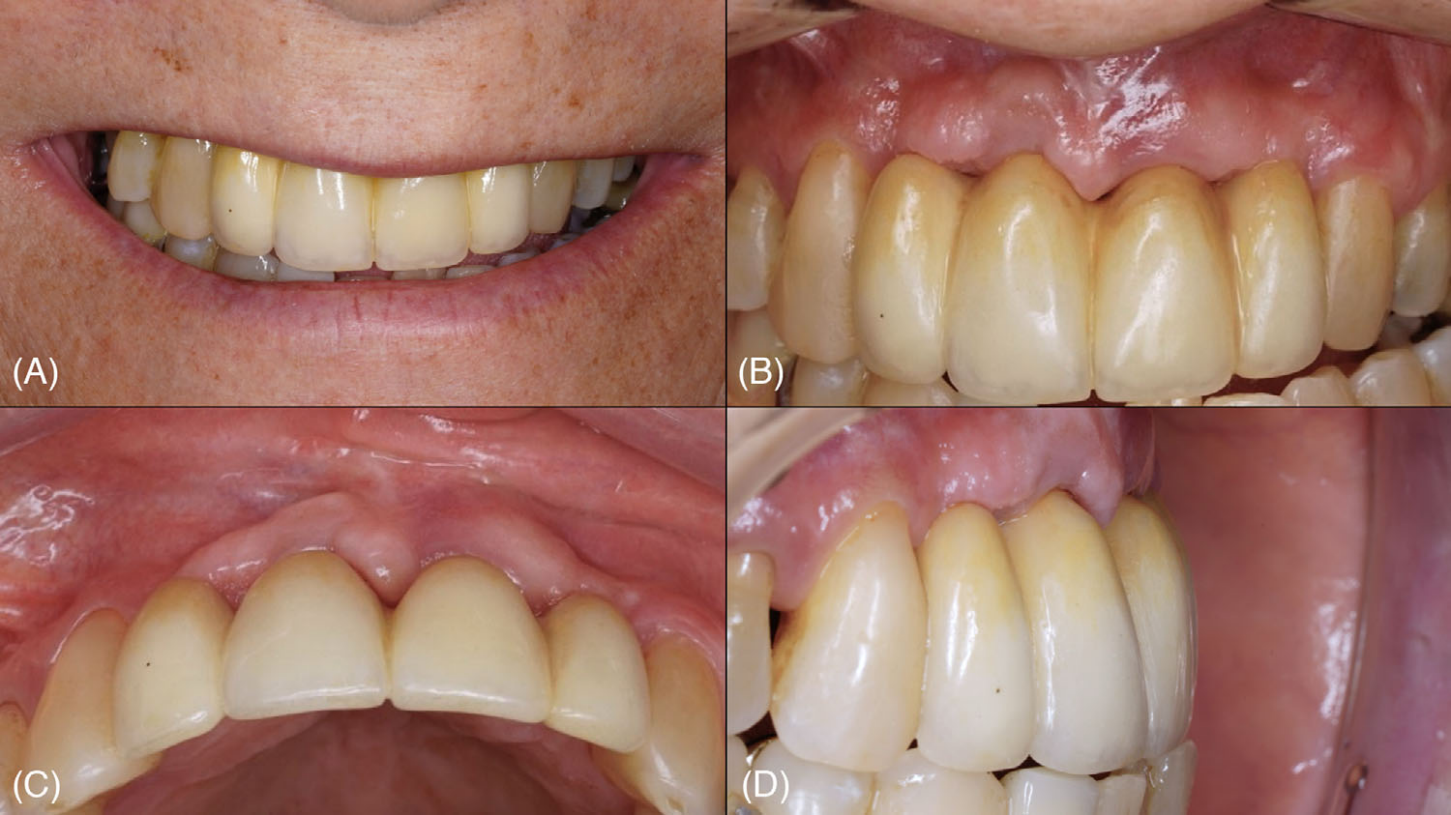
Case 1: Follow‐up aspects of the surgical site. (A) Patient with a low lip line during smiling. (B) Buccal view at 3 years postoperatively. (C) Occlusal view at 3 years postoperatively, note the thickness of soft tissue. (D) Lateral view at 3 years postoperatively, note the emergence profile.

At the 3‐year follow‐up (Figures 1C,D, and 3), a significant improvement in the peri‐implant phenotype was observed. PD at the buccal aspect was 2 mm, but a slight BOP was observed. The implant was fully covered by soft tissue, and the patient's complaint regarding mucosal transparency had been completely resolved. In addition, the patient was instructed to resume regular maintenance to maintain the results of the surgical procedure.

### Case 2

**FIGURE 4:**
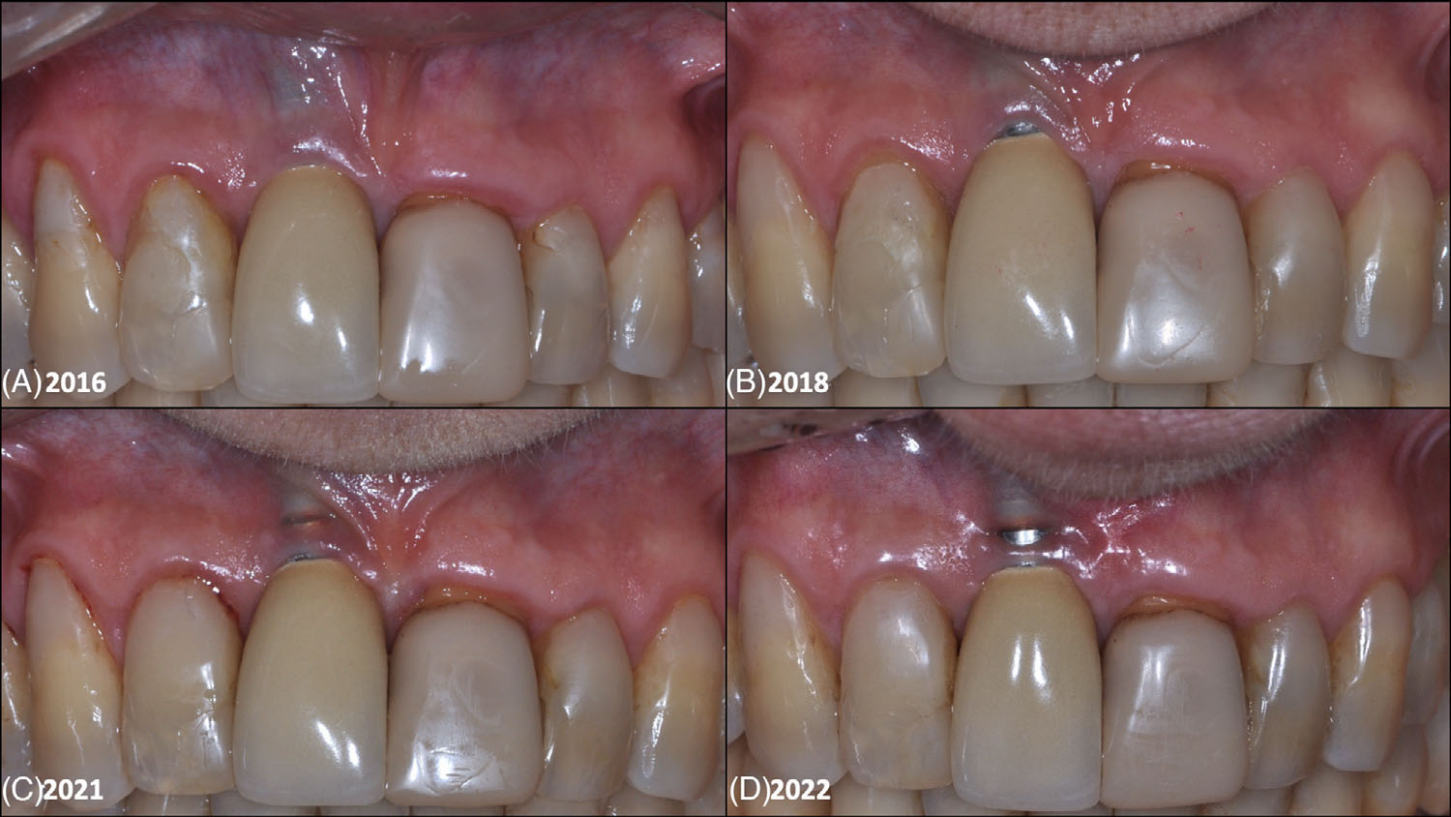
Case 2: Aspects of the progressive peri‐implant soft tissue defect. (A) Initial aspect of the buccal peri‐implant mucosa. (B) Aspect of the buccal peri‐implant mucosa at 2‐year follow‐up. (C) Aspect of the buccal peri‐implant mucosa at 5‐year follow‐up. (D) Aspect of the buccal peri‐implant mucosa at 6‐year follow‐up (baseline for surgery).

A 55‐year‐old female presented in 2016 with aesthetic concerns due to color alteration and mucosal recession on the buccal aspect of implant #8. Clinical examination showed adequate keratinized tissue near the mucosal margin but a thin adjacent mucosa. The patient was informed of risks such as further recession and implant exposure, but declined surgical intervention, opting for periodic monitoring. As there were no symptoms (pain, bleeding on probing) and the defect was not visible when smiling, clinical follow‐up was maintained during routine visits. She completed all dental treatments, including periodontal therapy, and remained under supportive care. Over time, progressive tissue thinning, mucosal darkening, and increased implant exposure were observed (Figure 4A–D). Despite earlier refusals, in 2022, after 6 years and significant worsening, the patient agreed to surgery, understanding the high risk of implant loss.

**FIGURE 5:**
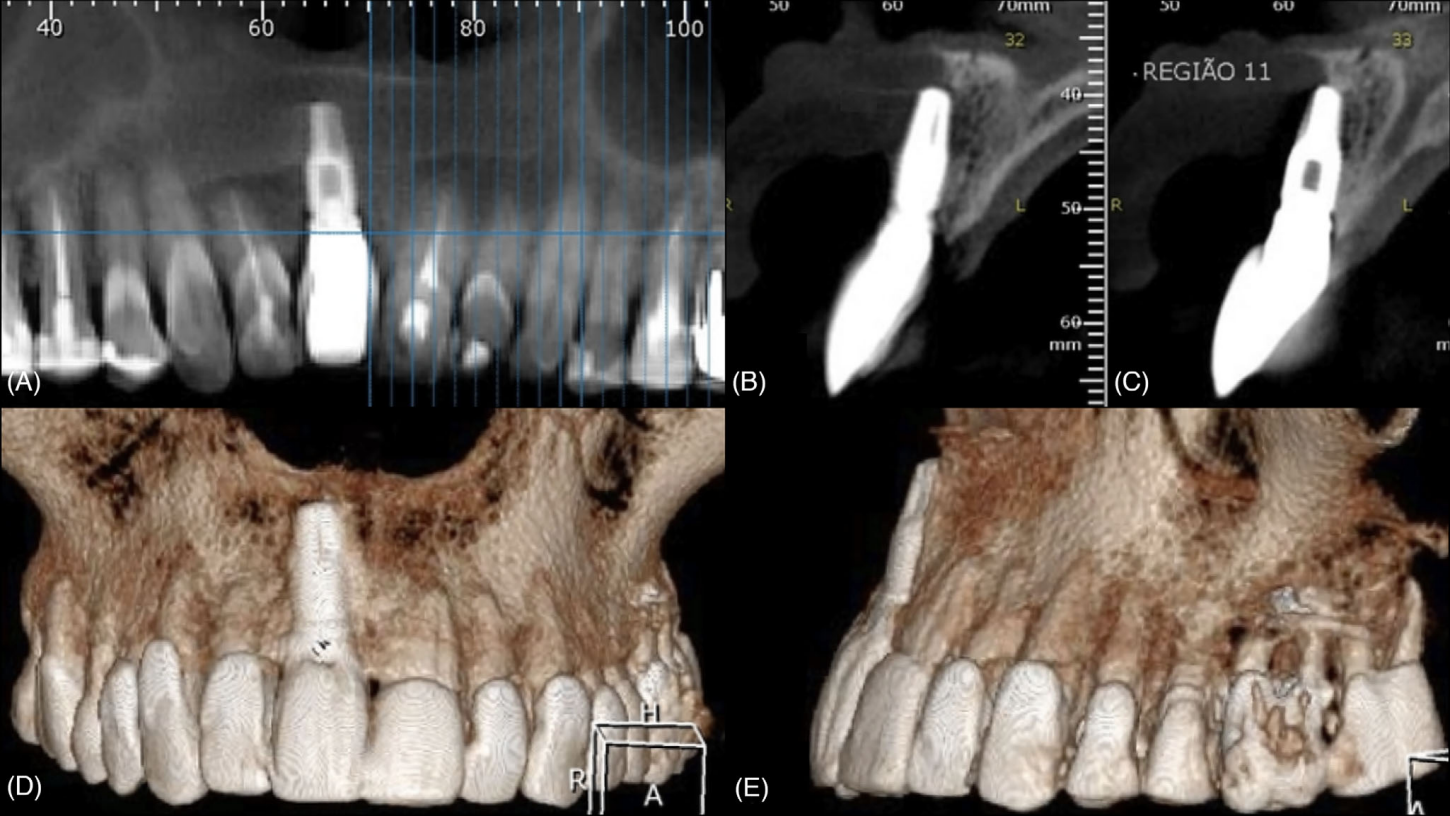
Case 2: Tomographic aspects of the implant position. (A) Panoramic slice evidencing the mesial‐distal position of the implant. (B–C) Sagittal cross‐sectional views showing the implant malposition and the complete absence of buccal bone wall. (D) Buccal view of the 3‐dimensional reconstruction of the tomographic images. (E) Lateral view of the 3‐dimensional reconstruction of the tomographic images.

Cone‐beam computed tomography (CBCT) showed the complete absence of buccal bone on the implant at #8, and that the implant was severely outside the bony envelope (Figure 5A–E).

**FIGURE 6:**
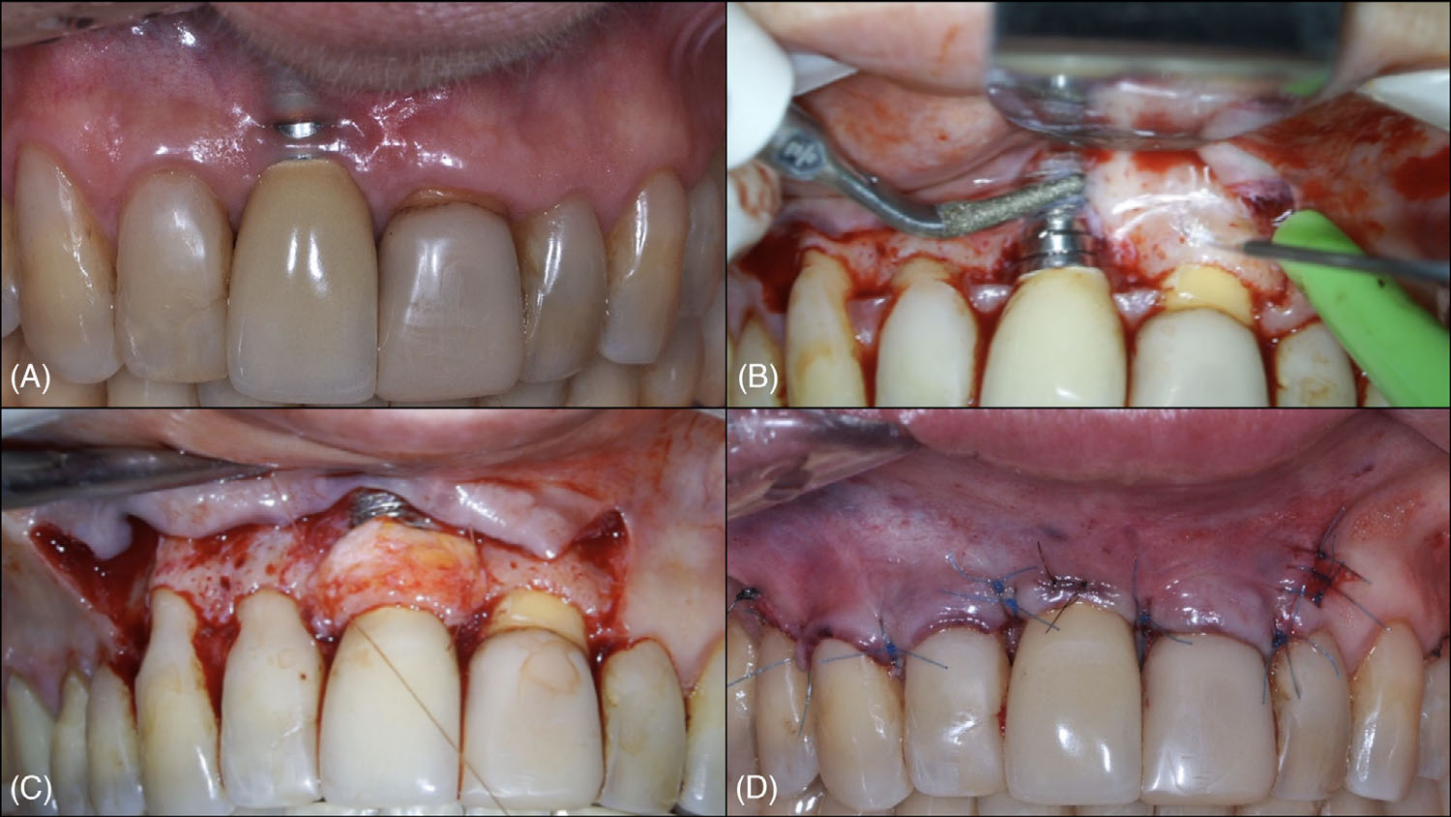
Case 2: Steps of the first surgical intervention. (A) Baseline aspect of the surgical site. (B) Implantoplasty with an ultrasonic diamond tip of higher‐grain size, after incision and flap elevation. (C) Stabilization of the CTG to the interproximal papillae. (D) Coronal advancement of the flap and stabilization with sutures 6.0 blue nylon and 7.0 black nylon at the fenestration.

**FIGURE 7:**
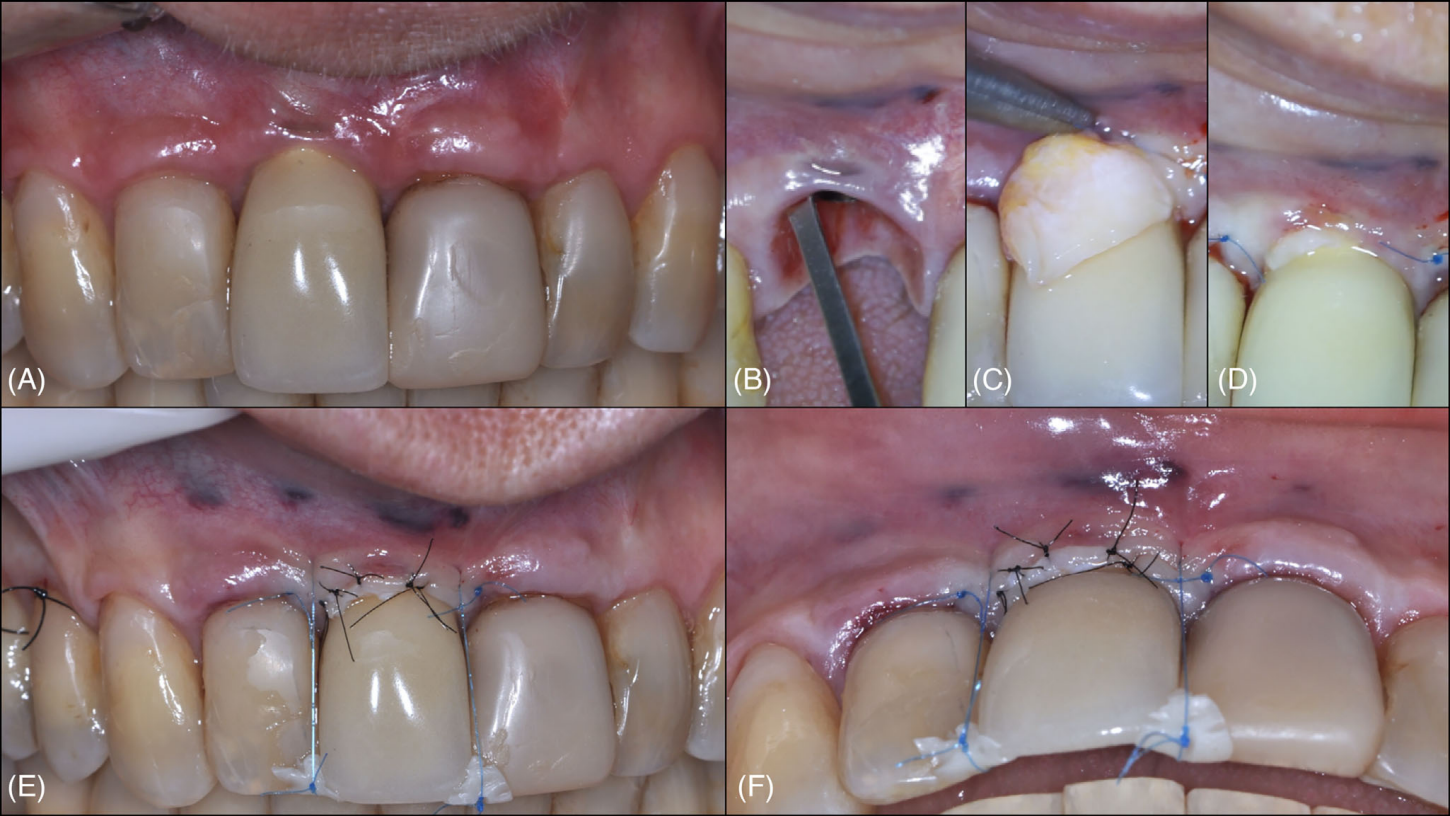
Case 2: Second surgical procedure. (A) Buccal view of the surgical site 3 months after the first intervention. (B) A microsurgical blade was used to split the buccal flap and to create a tunnel extending toward the adjacent teeth. (C) Insertion of the CTG into the mesial portion of the tunnel. (D) Interproximal stabilization of the CTG using 6.0 nylon single interrupted sutures. (E) Final buccal view immediately after surgery. Four 7.0 nylon single interrupted sutures were placed to secure the CTG to the overlying flap, enhancing its stability. Additionally, 6.0 nylon sling sutures were used to advance the flap coronally. (F) Final occlusal view immediately after surgery, demonstrating the increased buccal volume in the implant region of tooth #8.

The defect was managed with implantoplasty and CTG using an OM‐assisted approach. In the first surgery (Figure 6A–D), two vertical releasing incisions were made at distal #6 and distal #9, followed by a intrasulcular and a horizontal incision at the base of the papillae, which were preserved in the recipient bed and de‐epithelialized. A trapezoidal mucoperiosteal flap was carefully elevated to avoid tissue tearing near the implant. Implantoplasty was performed as described in Case 1 to reduce buccal volume and sharp threads, thereby minimizing the risk of soft tissue fenestration recurrence (Figure 6B). A palatal CTG was stabilized with 6.0 resorbable (Vicryl 6.0, Ethicon, Johnson & Johnson) single interproximal interrupted sutures (Figure 6C). The flap was coronally advanced and secured with 6.0 nylon (Nylon Blue, Techsuture) single interrupted sutures, and 7.0 nylon (Nylon Black, Techsuture) sutures were used to attempt closure of a soft tissue fenestration near the mucosal margin (Figure 6D). See Video S2 for further details. After 3 months, increased buccal soft tissue thickness and complete coverage of the implant shoulder were achieved. However, the mucosa remained thinner compared to adjacent teeth, especially at #9, and a small fenestration persisted (Figure 7A).

**FIGURE 8:**
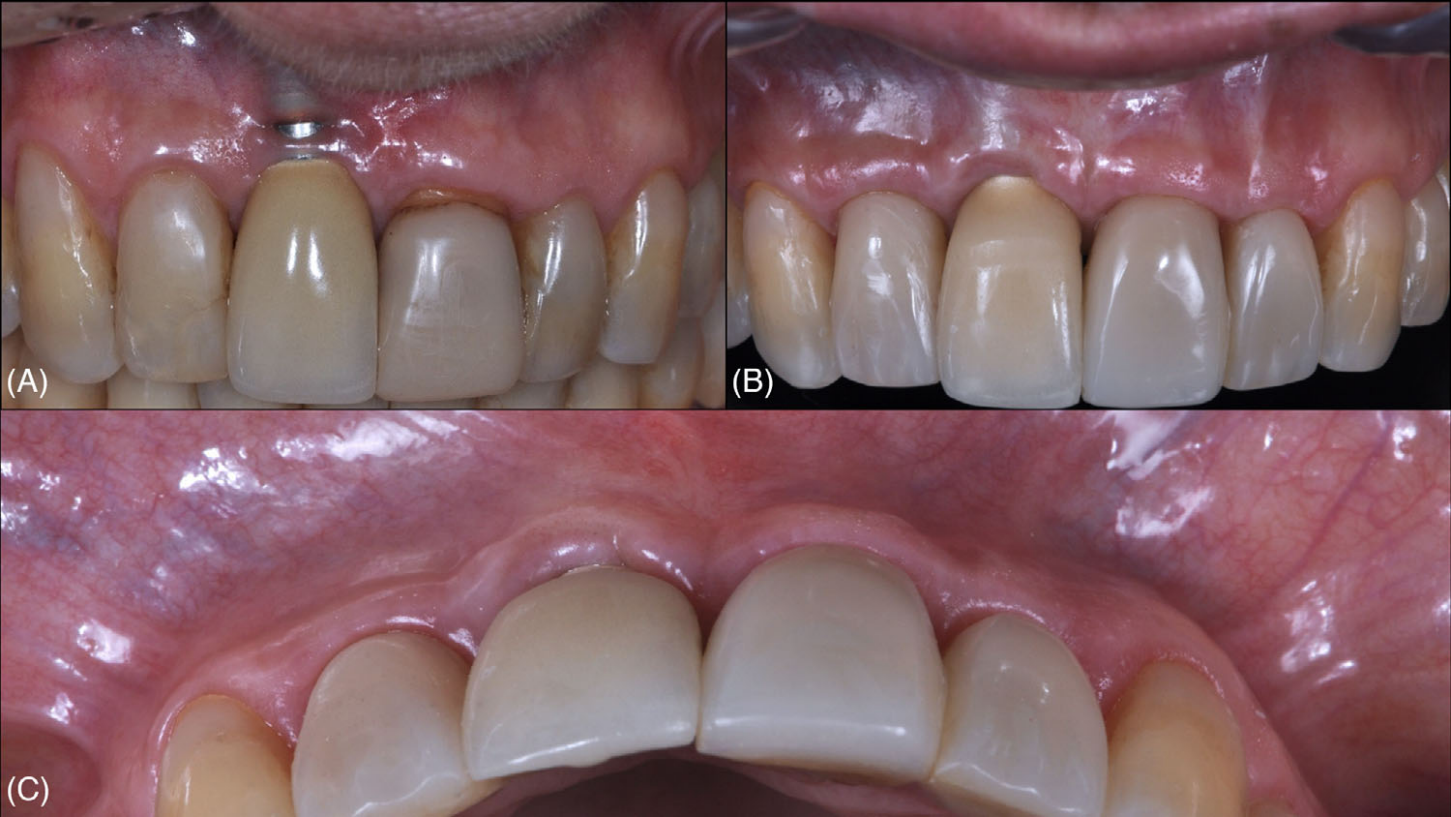
Case 2: Initial and final aspects following surgical interventions. (A) Aspect of the buccal peri‐implant mucosa at baseline. (B) Buccal view at 3 years after the first surgical intervention. (C) Incisal view at 3 years after the first surgical intervention.

Due to the postoperative characteristics after the first surgery, a second procedure was planned, 4 months after the first procedure, to enhance buccal soft tissue thickness and contour (Figure 7A–F). Using the OM, a flapless tunnel technique combined with CTG was performed. A microsurgical blade (Tunnel blade Arraia, Supremo Instrumentos Cirúrgicos) created a tunnel in the regions of #7, #8, and #9 (Figure 7B). The harvested CTG was inserted into the tunnel with a 6.0 nylon (Nylon Blue, Techsuture) suture starting at the mesial aspect of #9, engaging the graft, and returning near the entry point. The same was repeated at the mesial aspect of #7 (Figure 7C,D). Four single interrupted 7.0 nylon (Nylon Black, Techsuture) sutures stabilized the CTG to the buccal flap,^30^, ^31^ followed by 6.0 nylon (Nylon Blue, Techsuture) suspensory sutures to advance the flap coronally. These sutures were anchored to dental composite resin placed at the contact points between teeth #7 and #8, and between teeth #8 and #9 (Figure 7E,F). At the 3‐year follow‐up, significant soft tissue thickening and a natural contour that matched the adjacent teeth were observed (Figure 8). The PD at the buccal aspect was 2 mm without BOP. For a detailed description of the surgical procedure, refer to Video S3.

### Case 3

**FIGURE 9:**
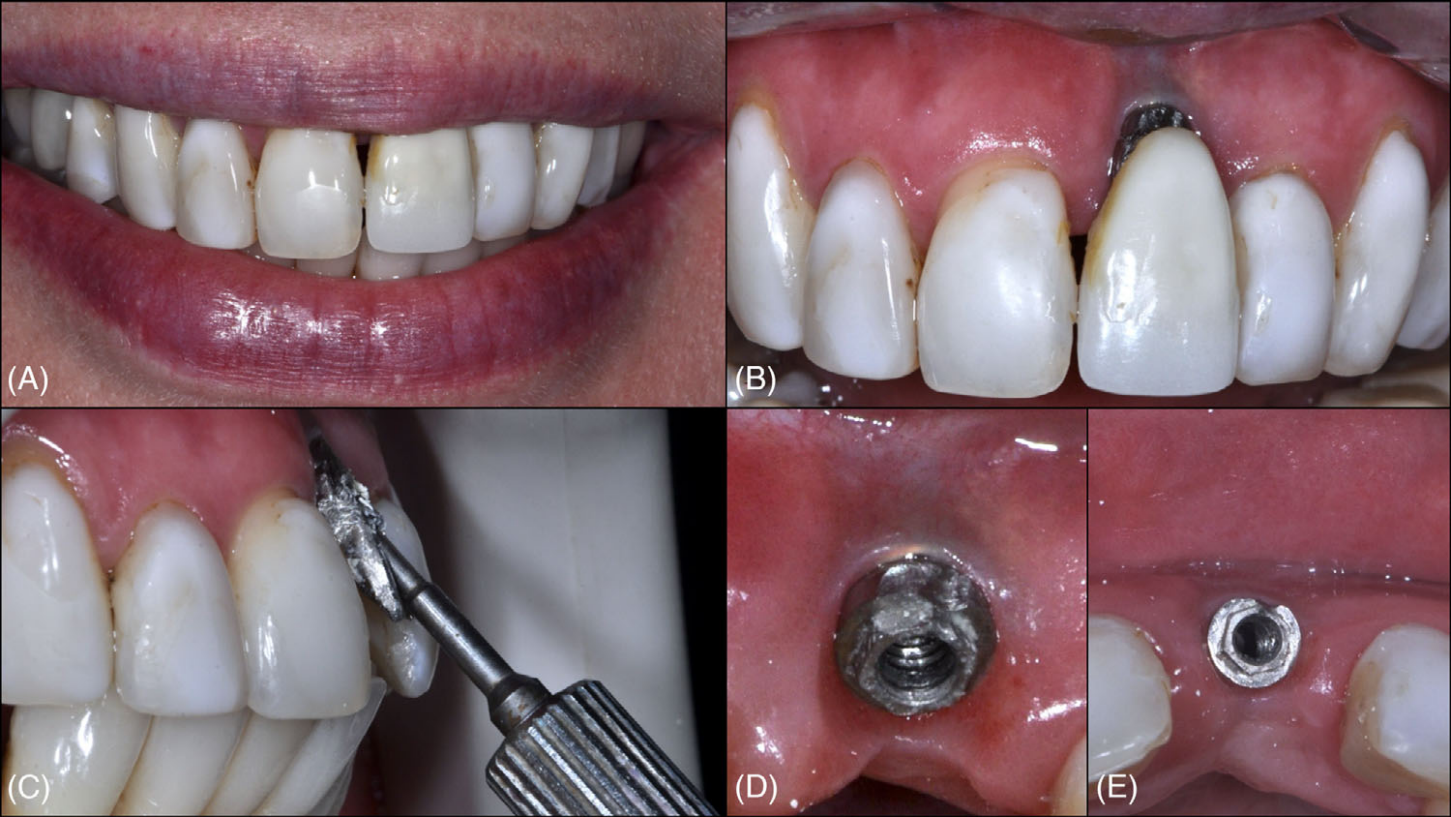
Case 3: Initial clinical situation. (A) Extraoral view of the patient's smile, characterized by a low smile line and the presence of a black space between the maxillary central incisors. (B) Frontal intraoral view showing peri‐implant soft tissue discoloration, volume deficiency, and apical migration of the mucosal margin. (C) Lateral intraoral view after removal of the crown, with the abutment still in place, highlighting the suboptimal three‐dimensional positioning of the implant in the buccolingual direction. (D) Occlusal intraoral view after removal of both the crown and abutment, revealing the thin peri‐implant soft tissue and clinical evidence of inadequate implant positioning. (E) Occlusal view clearly illustrating the buccolingual malposition of the implant, contributing to the deficiency in buccal tissue volume.

**FIGURE 10:**
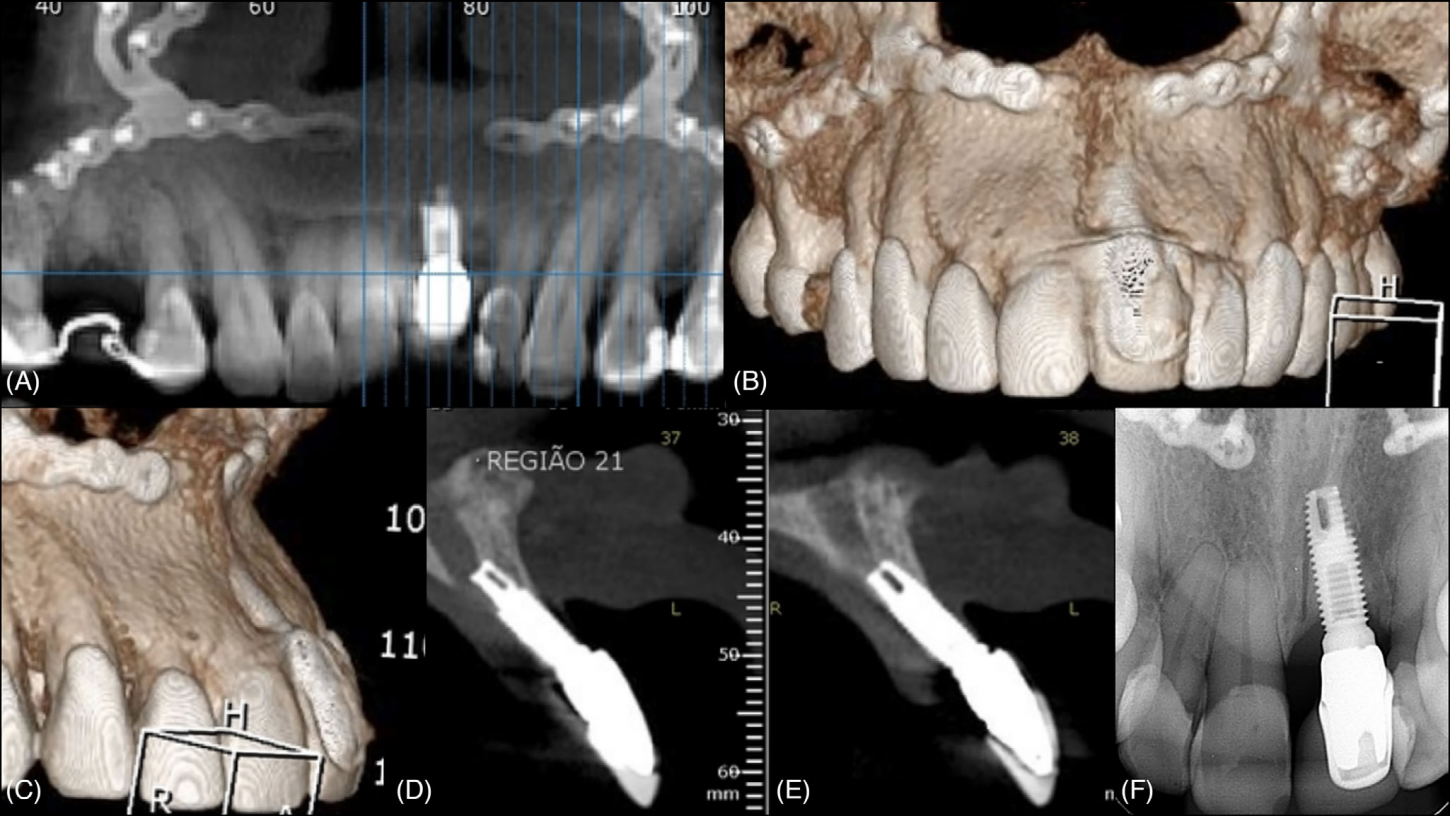
Case 3: Initial radiographic evaluation. (A) Panoramic reconstruction showing the implant in the region of tooth #9. (B) 3D reconstruction reveals the implant's buccopalatal volume and the significant bone deficiency in the region. The presence of fixation plates from a previous orthognathic surgery is also noted. (C) A lateral view of the 3D reconstruction confirms the vestibular positioning of the implant and the associated bone deficiencies. (D–E) Sagittal cross‐sectional views demonstrating the buccal angulation of the implant, associated with both buccal and palatal bone loss, and its proximity to the nasopalatine canal. Additionally, the apical bone morphology appears unfavorable, with limited dimensions. (F) Periapical radiograph showing that, despite the absence of buccal and palatal bone plates, the interproximal bone levels remain satisfactory.

A 42‐year‐old female patient presented dissatisfied with an implant‐supported rehabilitation performed 12 years earlier. Clinical examination revealed a malpositioned implant in the apicocoronal and buccopalatal dimensions, causing volume deficiency and mucosal discoloration at site #9, associated with over‐contoured, poorly colored composite restorations, gingival recessions, and a thin periodontal phenotype on adjacent teeth (Figure 9A–C). A CBCT confirmed these findings (Figure 10). Additionally, the implant shoulder was damaged, preventing proper abutment adaptation and promoting plaque accumulation (Figure 9D,E). PD at the center of the buccal aspect was 2 mm, and BOP was presented.

**FIGURE 11:**
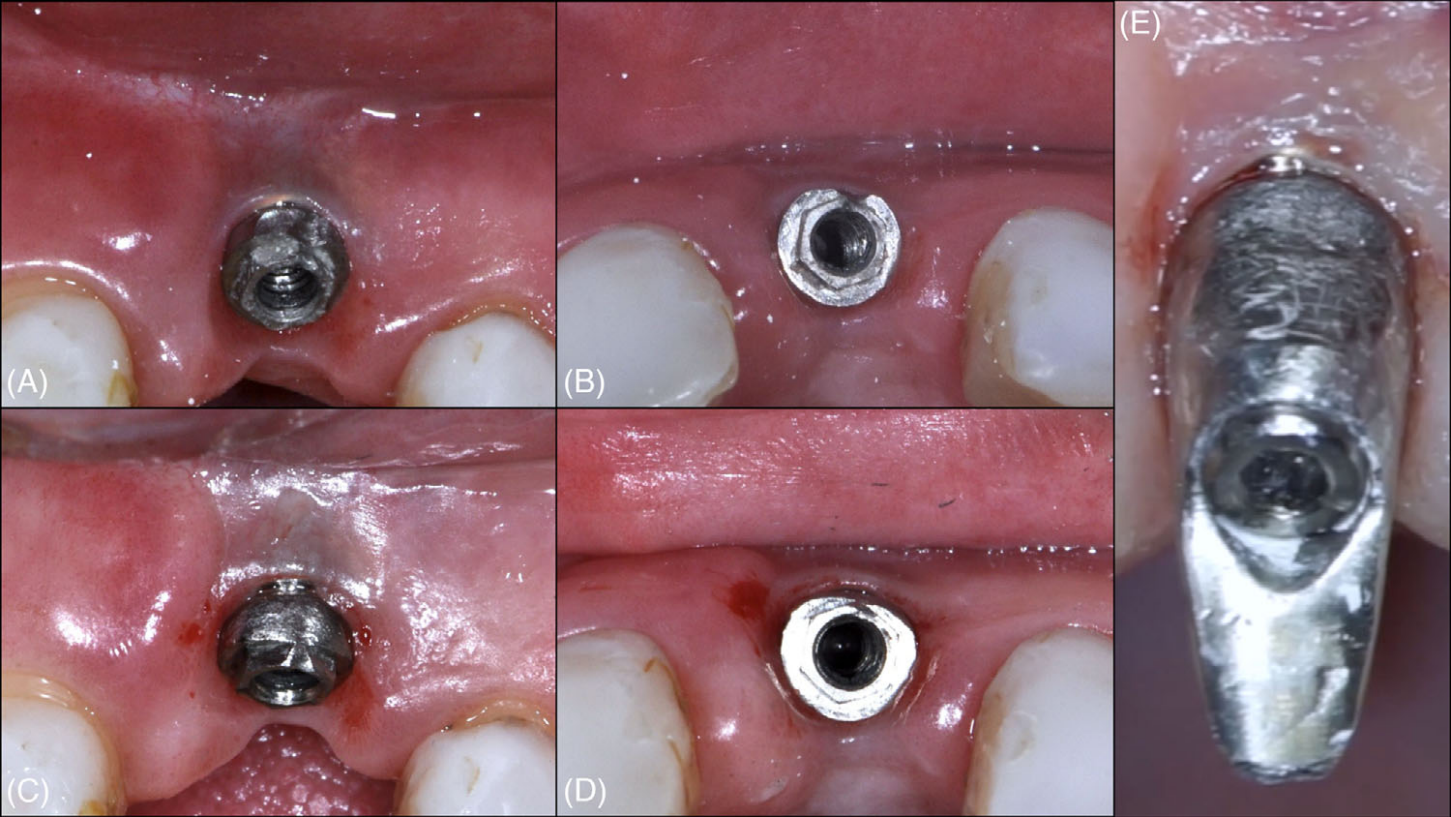
Case 3: Clinical views before and after non‐surgical implantoplasty. (A–B) Baseline occlusal views demonstrating the peri‐implant mucosa and implant exposure in the region of tooth #9. (C–E) Views after non‐surgical implantoplasty, performed without flap elevation using ultrasonic diamond and metallic tips along with an optical magnifier.

The treatment plan included buccal implantoplasty to adapt the abutment, reduce implant volume, and improve the predictability of a CTG. Initially, implantoplasty was performed without flap elevation, using diamond burs (FG 1016, KG Sorensen), ultrasonic tips (E7D Helse Ultrasonic, E6D Helse Ultrasonic, P10 Helse Ultrasonic), and an OM (Advanced Decius Microscope, DF Vasconcelos) to minimize trauma. This reshaped the implant body and implant abutment, creating a soft tissue depression for future grafting (Figure 11, Video S4).

**FIGURE 12:**
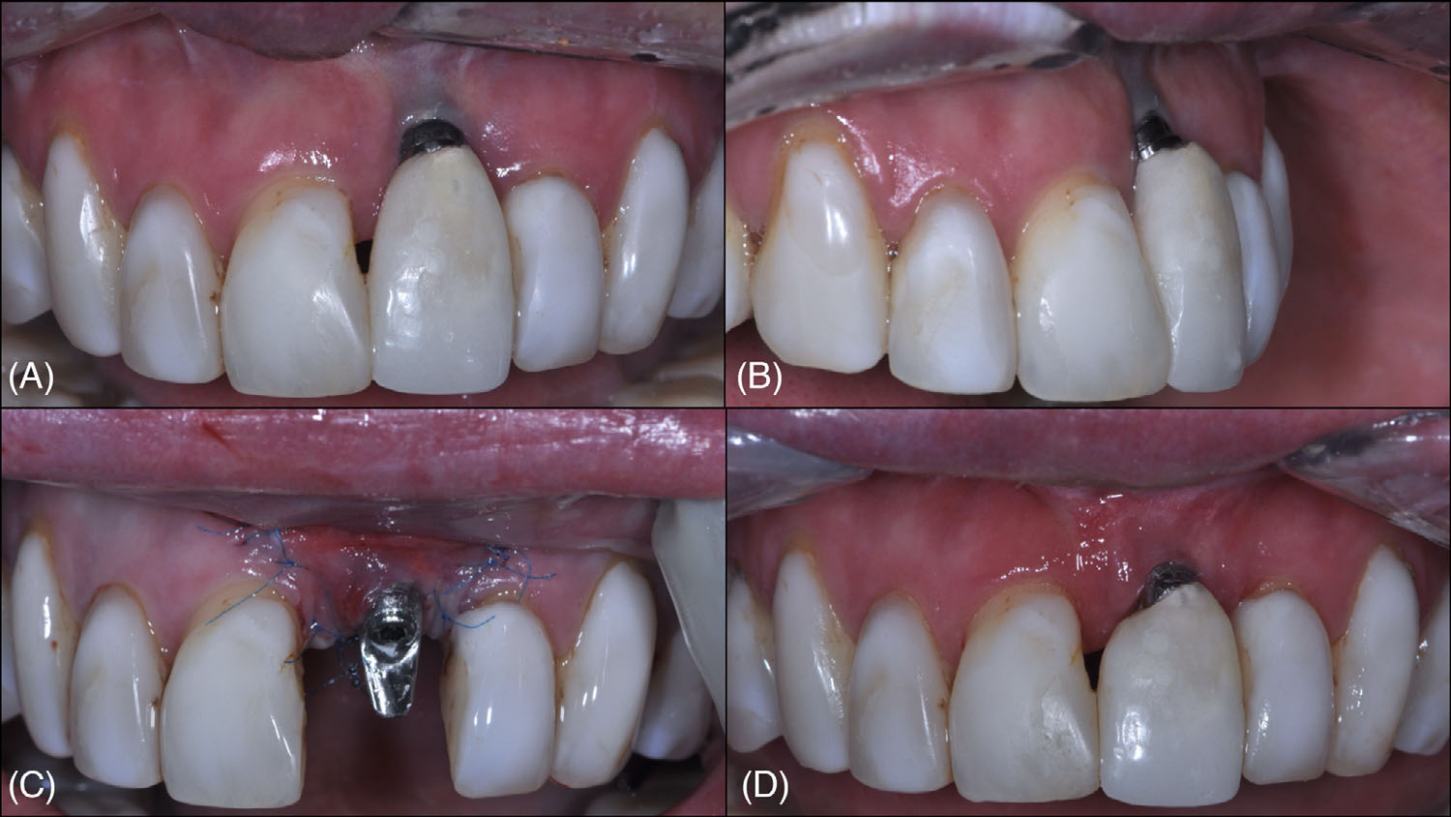
Case 3: First surgical intervention. (A) Baseline frontal view of the peri‐implant region. (B) Baseline lateral view showing soft tissue recession and deficient volume. (C) Immediate postoperative view before provisional crown cementation. A coronally advanced flap with two vertical releasing incisions was performed in combination with a connective tissue graft. (D) Postoperative follow‐up at 1 month demonstrating improved peri‐implant soft tissue contour and conversion to a thick tissue phenotype.

After 2 months, a surgical procedure was planned that involved a coronally advanced flap combined with a CTG (Figure 12, Video S5). During this intervention, an implantoplasty refinement was performed before and after flap elevation. After two vertical releasing incisions, a full‐thickness flap was reflected, and further implantoplasty refinement was completed. A graft from the palate was stabilized with 6.0 nylon (Nylon Blue, Techsuture) sutures, and the flap was coronally advanced.

**FIGURE 13:**
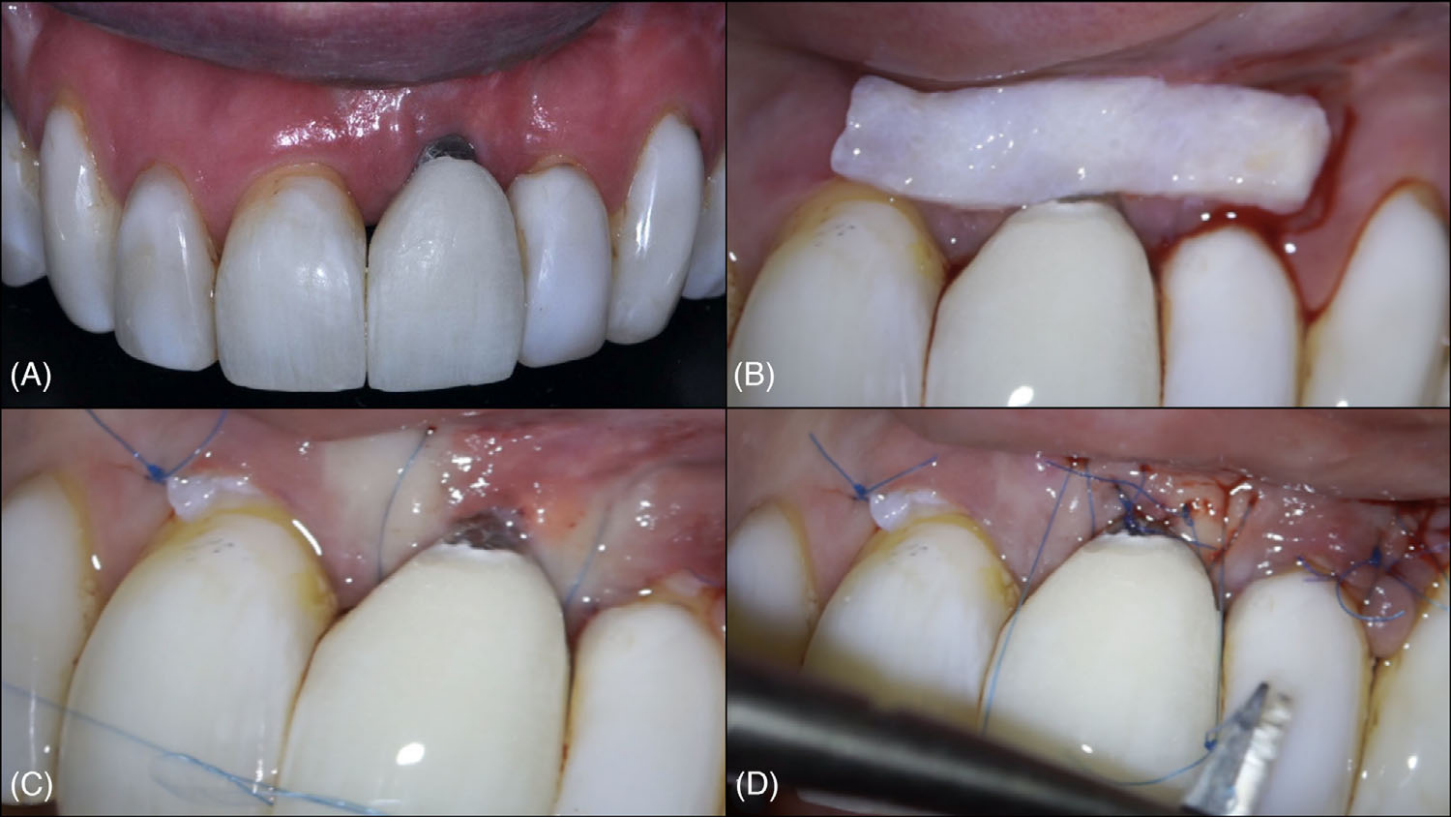
Case 3: Second surgical procedure. (A) Baseline view following 2 months of healing after the first intervention. (B) A tunneling coronally advanced flap technique was performed, with a minimal vertical releasing incision at the distal aspect of tooth #10, followed by a full‐thickness flap that extended to the region of tooth #8. A connective tissue graft harvested from the lateral palate with a corresponding area of three teeth. (C) The graft was inserted into the prepared tunnel and was stabilized using a horizontal mattress suture at the distal of tooth #8 and a single interrupted suture at the vertical incision distal to tooth #10. (D) The flap was advanced coronally and secured with 6.0 sling sutures engaging both the graft and the flap at region #9. The vertical releasing incision was closed with 7.0 nylon sutures, and additional microsutures were used to ensure intimate adaptation of the graft to the underlying tissues.

**FIGURE 14:**
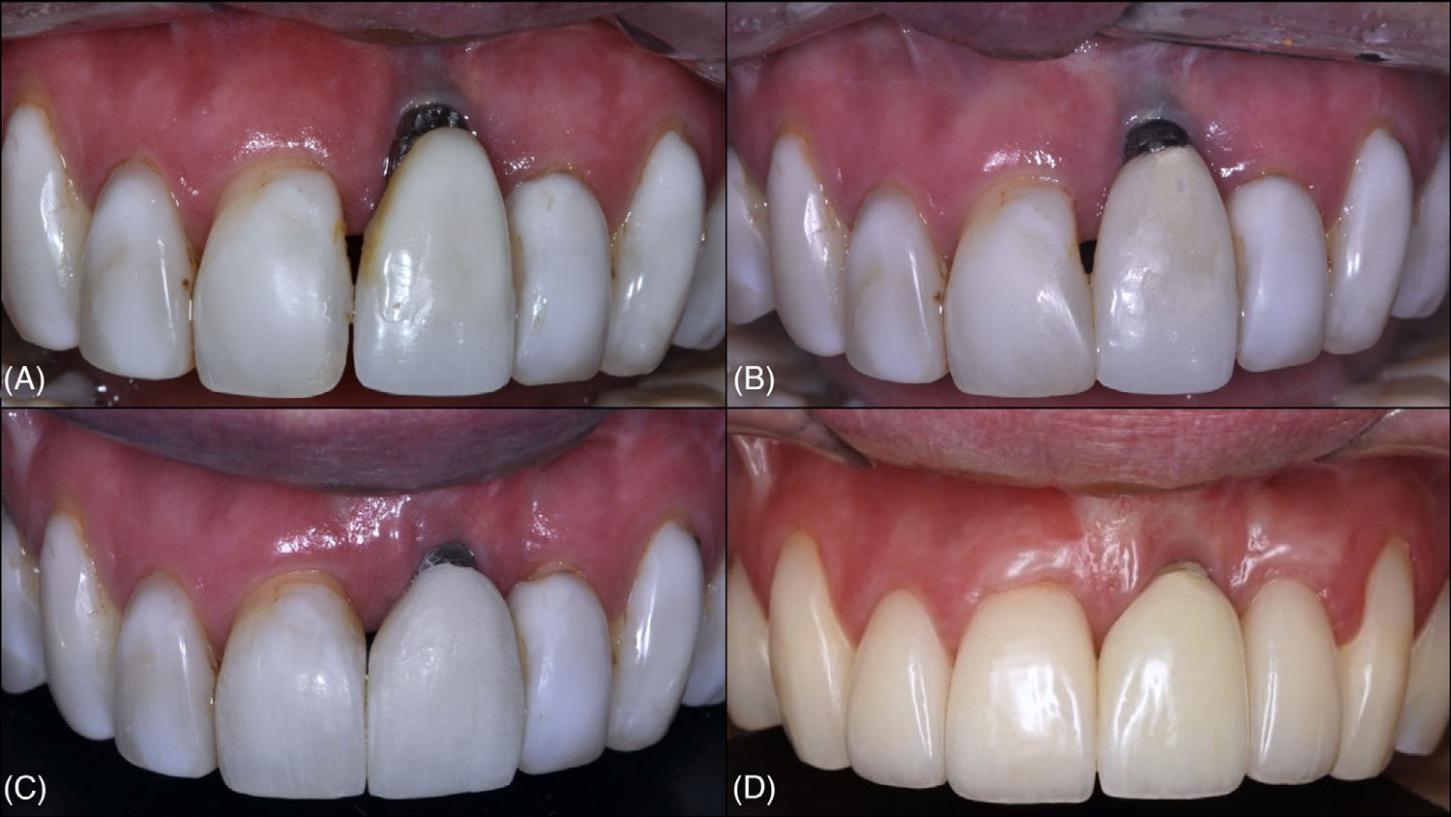
Case 3: Frontal view showing the clinical progression following surgical interventions. (A) Baseline image before any surgical treatment. (B) Two months after the non‐surgical implantoplasty procedure. (C) Three months after the first surgical intervention. (D) Six months after the second surgical procedure, revealing a significant improvement in the peri‐implant soft tissue phenotype.

**FIGURE 15:**
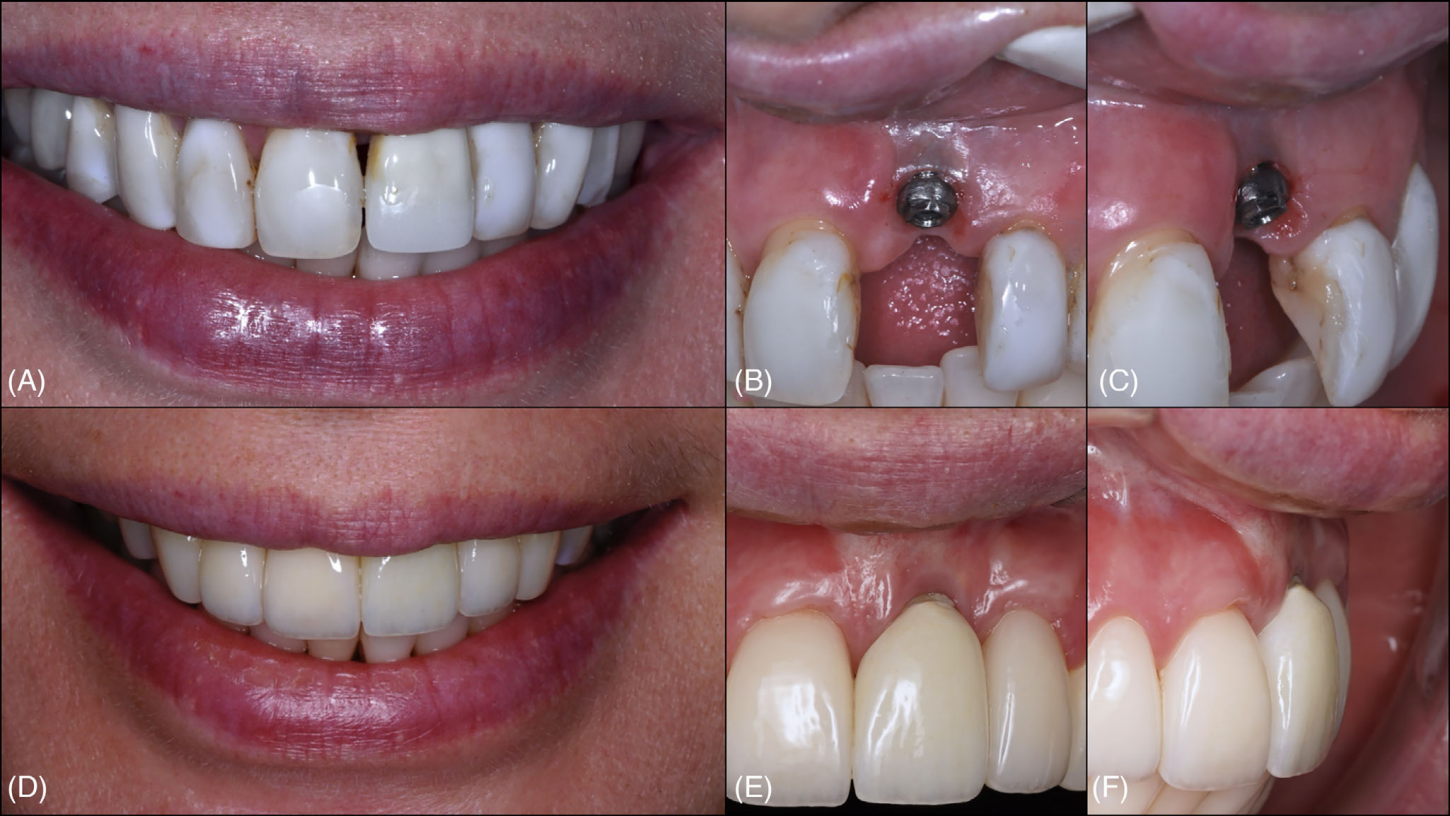
Case 3: Initial and final clinical appearance following surgical procedures. (A) Initial smile view. (B) Initial frontal intraoral view. (C) Initial lateral intraoral view. (D) Smile view at 1 year following the first surgical intervention. (E) Final frontal view. (F) Final lateral view.

At the 3‐month follow‐up, soft tissue thickening was evident. However, due to the severe malposition, a second connective tissue graft was indicated (Figure 13, Video S6). A tunneling coronally advanced flap was performed through a minimal vertical incision at distal #10, extending to #8. Another graft was harvested and placed, stabilized with a horizontal mattress suture at distal #8 and an interrupted suture at distal #10. The flap was coronally advanced using sling sutures around #9 and 7.0 nylon (Nylon Black, Techsuture) interrupted sutures were placed close to the margin for the final adjustment of the CTG position. Figure 14A–D illustrates the clinical progression of the case, from the initial presentation through the non‐surgical implantoplasty, the first surgical intervention, and the second surgical procedure. At the 6‐month follow‐up after the last surgical procedure, a substantial increase in soft tissue thickness was observed, yielding a more natural contour at site #9 and significantly reducing the visibility of the grayish hue (Figure 15). The PD at the final follow‐up was 2 mm without BOP.

## RESULTS

Overall, all procedures successfully preserved implants in highly compromised conditions. The microsurgical approach was crucial for precise, minimally traumatic interventions, particularly during implantoplasty, allowing safe and accurate recontouring without damaging adjacent soft and hard tissues  . In all cases, dark discoloration was eliminated, restoring the normal gingival color, and implant shoulder exposures were resolved, improving hygiene and maintenance. In general, the latest follow‐up showed the absence of BOP and probing depths ≤ 4 mm (Table S1).

Specifically, in Case 1, a recession reduction of 2 mm was observed, covering exposed threads and the implant shoulder, along with a significant increase in thickness, ensuring long‐term stability. In Case 2, buccal fenestration closure and a 1.5 mm recession reduction were achieved. Implantoplasty removed sharp angles that likely contributed to the fenestration, while soft tissue thickening and color improvement further enhanced the case prognosis.

In Case 3, an implant with severe buccal and crestal bone loss was preserved. OM‐assisted implantoplasty recontoured the damaged shoulder, enabling prosthetic rehabilitation without compromising adjacent tissues. When compared to the preoperative condition, approximately 2 mm of recession reduction was achieved, in addition to increased tissue thickness, providing critical protection in an area previously subjected to extensive surgical procedures.

## DISCUSSION

This case series illustrates clinical situations where OM‐assisted soft tissue grafting and implantoplasty enabled implant preservation in scenarios that would otherwise require removal and extensive reconstruction. In all cases, implant removal would have led to greater morbidity, higher risk to adjacent structures, and, particularly in Cases 2 and 3, an unpredictable surgical prognosis due to implant dimensions and delicate adjacent structures. Although all cases involved maxillary central incisors, patient priorities centered on minimally invasive solutions rather than perfect esthetic outcomes. In contrast with the conventional approach, that is, implant removal followed by hard and soft tissue grafting,^10^, ^32^ this report suggests that implantoplasty associated with CTG may offer stable, satisfactory results without implant explantation. Despite the positive results, it is important to emphasize that these are borderline situations, with implants lacking buccal bone and positioned outside the bony housing. From an esthetic standpoint, the present approach can be considered limited, but implant removal also presents significant risks and challenges and may not achieve ideal esthetics either. Our approach was to inform the patients of all the advantages, disadvantages, and limitations of all treatment options. The main advantages of the present approach are its less invasive nature and lower morbidity, as well as the creation of a soft tissue condition that allows for the maintenance of peri‐implant health over time. The benefits of soft tissue augmentation, especially increasing buccal thickness, are well‐documented for supporting peri‐implant health and esthetic stability.^33^ This aligns with other literature recommendations that suggest an association between soft tissue and restorative procedures, aside from implant removal.^34^

Despite previous contraindications for implantoplasty in esthetic areas,^35^, ^36^ favorable outcomes were achieved, potentially influenced by the use of an OM, which enhanced surgical precision in biologically challenging scenarios.^17^ Considering the complexity of this type of procedure in the esthetic zone, it is speculated that medium (8–16×) to high (>16×) levels of magnification, both compatible with OM magnification, appear to be fundamental in improving implantoplasty precision, minimizing prosthetic or tissue damage, even when prostheses remain in place. It also helps determine the extent of the implantoplasty area to be performed, which may vary according to each clinical scenario. In the presence of infection, such as Cases 1 and 2, implantoplasty is usually performed on the buccal aspect of supracrestal threads. Particularly in Case 2, the objectives included a buccal reduction of the implant shoulder and the removal of the supracrestal threads that had caused tissue damage and soft tissue fenestration. Given that the implant had a relatively large diameter, the risk of fracture was low. In the absence of disease, implantoplasty in the esthetic zone can be limited to the supra mucosal threads, and this was the scenario of Case 3. However, the implant of Case 3 had a damaged platform and a smaller implant. The purpose of the implantoplasty in this case was to create a smooth area with an adequate transition between the implant shoulder and the abutment, preventing a step and plaque accumulation. The procedure was aimed at surface smoothing, but the risk of implant weakening was fully discussed with the patient. This aspect is also directly related to the choice of flap design. In situations where access to the implant threads is needed or there is risk of soft tissue damage, flap design may require vertical incisions, and CAF may be the appropriate approach instead of a tunnel approach.

The use of OM also contributed to more effective connective tissue graft adaptation and wound stabilization, which can make the difference particularly in complex anatomical contexts.^18^ Additionally, the microsutures placed at the soft tissue margin provide additional immobilization of the graft while reducing the thickness of the clot layer between the graft and the flap.^30^, ^31^ Such conditions contribute to a predictable tissue gain, as observed in our cases. For these reasons, the decision to intentionally leave the tissue partially exposed was aimed at optimizing the gain of keratinized tissue, as already described by the periodontal surgery literature,^37^ with the microsurgical approach reinforcing the safety of this conduct.

The adoption of magnification in implantology reflects broader trends in other dental specialties, where OM use correlates with better healing, less surgical trauma, improved esthetic results, and greater operator comfort.^26^, ^38^ Although OM requires a significant financial investment and a steep learning curve,^39^ its clinical advantages may outweigh these limitations, especially in complex cases. Dental loupes, while offering less magnification, remain a practical alternative with demonstrated benefits.^16^, ^40^

Furthermore, precise implantoplasty may reduce the risk of mechanical complications associated with excessive material removal, an important consideration for narrow implants.^4^ The possibility of achieving a smoother implant surface with OM assistance, as observed in this case series, echoes findings in restorative dentistry where magnification improves preparation precision.^41^

Another point of interest is the role of implantoplasty in reducing the avascular surface area, facilitating better integration of soft tissue grafts, analogous to the concept of odontoplasty in root coverage procedures.^42^ This strategy may be valuable for managing buccally positioned implants in esthetic areas, although it cannot be generalized to all cases without careful assessment.^10^, ^43^

It is worth mentioning that the consistent benefits of the described techniques shall be interpreted with caution, mainly due to the retrospective non‐comparative nature of this case study. and because of the skill and training level of the surgeon, which is critical when using the OM. On the other hand, while the current literature on implantoplasty's role in esthetic areas remains limited, the findings presented suggest that, when combined with soft tissue grafting and performed under magnification, it may represent a viable alternative to explantation in select scenarios. These promising results pave the way toward future prospective controlled randomized designs to better define indications, limitations, and long‐term outcomes associated with this approach.

## CONCLUSION

Microsurgery combined with implantoplasty and connective tissue grafting may represent an effective alternative for managing supraosseous or exposed implant threads caused by peri‐implant disease or poor implant positioning. Magnification combined with advanced skills for surgeons enhance implant surface smoothing and precise flap and graft handling, contributing to more predictable and stable long‐term outcomes.

## AUTHOR CONTRIBUTIONS

**João Batista Cesar Neto**: Conceptualization; formal analysis; investigation; methodology; resources; writing—original draft preparation; writing—review & editing. **Rafael Lazarin, Henrique Rinaldi Matheus, Emerson Santiago**: Formal analysis; writing—original draft preparation; writing—review & editing. **Giuseppe A. Romito**: Writing—review & editing.

## CONFLICT OF INTEREST STATEMENT

The authors declare no conflicts of interest.

## Supporting information




**Video 1**. Case 1 – Surgical procedure.

**Video 2**. Case 2 – First surgical procedure.

**Video 3**. Case 2 – Second surgical procedure.

**Video 4**. Case 3 – Non‐surgical implantoplasty.

**Video 5**. Case 3 – First surgical procedure.

**Video 6**. Case 3 – Second surgical procedure.

**Supplementary Table 1**. Peri‐implant clinical parameters.
MB: mesiobuccal, B: buccal, DB: distobuccal, MP: mesiopalatal, P: palatal, DP: palatal, PI: plaque index (yes/no), BOP: bleeding on probing (yes/no), PD: probing depth in mm, MR: mucosal recession in mm, KTW: keratinized tissue width in mm.

## Data Availability

The data that support the findings of this study are available on request from the corresponding author. The data are not publicly available due to privacy or ethical restrictions.
