# Supplementary material for: Implantoplasty combined with soft tissue grafting for the management of complex cases: A microsurgical approach
Source: Clin Adv Periodontics. 2026 Apr 15;16(Suppl 1):S97–S110. doi: 10.1002/cap.70037 (PMC13080409; doi:10.1002/cap.70037)
Supplement: Supplementary file 7 — Supplementary Table 1. Peri‐implant clinical parameters. MB: mesiobuccal, B: buccal, DB: distobuccal, MP: mesiopalatal, P: palatal, DP: palatal, PI: plaque index (yes/no), BOP: bleeding on probing (yes/no), PD: probing depth in mm, MR: mucosal recession in mm, KTW: keratinized tissue width in mm. [file CAP-16-S97-s002.docx]

**Table 1.** Peri-implant clinical parameters

|  | **Baseline** | | | | | | **Last follow-up** | | | | | |
| --- | --- | --- | --- | --- | --- | --- | --- | --- | --- | --- | --- | --- |
|  | **Buccal** | | | **Palatal** | | | **Buccal** | | | **Palatal** | | |
|  | **MB** | **B** | **DB** | **MP** | **P** | **DP** | **MB** | **B** | **DB** | **MP** | **P** | **DP** |
| **Case 1 - Implant # 8** | | | | | | | | | | | | |
| PI | Yes | Yes | No | No | No | No | No | No | No | No | No | No |
| BOP | Yes | Yes | No | No | No | No | No | Yes | No | No | No | No |
| PD | 4 | 2 | 3 | 4 | 3 | 3 | 3 | 2 | 3 | 3 | 3 | 3 |
| MR | - | 2 | - | - | - | - | - | 0 | - | - | - | - |
| KTW | - | 2 | - | - | - | - | - | 4 | - | - | - | - |
| **Case 1 - Implant #9** | | | | | | | | | | | | |
| PI | Yes | No | No | No | No | No | No | No | No | No | No | No |
| BOP | Yes | Yes | No | No | No | No | Yes | No | No | Yes | No | No |
| PD | 3 | 3 | 3 | 3 | 4 | 4 | 3 | 3 | 3 | 3 | 3 | 3 |
| MR | - | 0 | - | - | - | - | - | 0 | - | - | - | - |
| KTW | - | 3 | - | - | - | - | - | 4 | - | - | - | - |
| **Case 2 – Implant #8** | | | | | | | | | | | | |
| PI | No | Yes | No | No | No | No | No | No | No | No | No | No |
| BOP | Yes | No | No | No | No | No | No | No | No | No | No | No |
| PD | 2 | 1 | 3 | 3 | 2 | 3 | 2 | 2 | 3 | 3 | 2 | 3 |
| MR | - | 2 | - | - | - | - | - | 0.5 | - | - | - | - |
| KTW | - | 0 | - | - | - | - | - | 1.5 | - | - | - | - |
| **Case 3 – Implant #9** | | | | | | | | | | | | |
| PI | No | No | Yes | No | No | No | No | No | No | No | No | No |
| BOP | Yes | Yes | Yes | No | No | Yes | No | No | No | No | No | No |
| PD | 4 | 2 | 4 | 4 | 4 | 4 | 2 | 2 | 3 | 3 | 3 | 3 |
| MR | - | 3 | - | - | - | - | - | 1 | - | - | - | - |
| KTW | - | 0 | - | - | - | - | - | 2 | - | - | - | - |

MB: mesiobuccal, B: buccal, DB: distobuccal, MP: mesiopalatal, P: palatal, DP: palatal, PI: plaque index (yes/no), BOP: bleeding on probing (yes/no), PD: probing depth in mm, MR: mucosal recession in mm, KTW: keratinized tissue width in mm.
